# Review: divergent selection for residual feed intake in the growing pig

**DOI:** 10.1017/S175173111600286X

**Published:** 2017-01-25

**Authors:** H. Gilbert, Y. Billon, L. Brossard, J. Faure, P. Gatellier, F. Gondret, E. Labussière, B. Lebret, L. Lefaucheur, N. Le Floch, I. Louveau, E. Merlot, M.-C. Meunier-Salaün, L. Montagne, P. Mormede, D. Renaudeau, J. Riquet, C. Rogel-Gaillard, J. van Milgen, A. Vincent, J. Noblet

**Affiliations:** 1 GenPhySE, INRA, INP, ENSAT, Université de Toulouse, 31326 Castanet-Tolosan, France; 2 GenESI, INRA, 17700 Surgères, France; 3 PEGASE, INRA, Agrocampus Ouest, 35590 Saint-Gilles, France; 4 QuaPA, INRA, 63122 Saint Genès-Champanelle, France; 5 GABI, INRA, AgroParisTech, Université Paris-Saclay, 78350 Jouy-en-Josas Cedex, France

**Keywords:** pig, genetics, selection, feed efficiency, residual feed intake

## Abstract

This review summarizes the results from the INRA (Institut National de la Recherche Agronomique) divergent selection experiment on residual feed intake (RFI) in growing Large White pigs during nine generations of selection. It discusses the remaining challenges and perspectives for the improvement of feed efficiency in growing pigs. The impacts on growing pigs raised under standard conditions and in alternative situations such as heat stress, inflammatory challenges or lactation have been studied. After nine generations of selection, the divergent selection for RFI led to highly significant (*P*<0.001) line differences for RFI (−165 g/day in the low RFI (LRFI) line compared with high RFI line) and daily feed intake (−270 g/day). Low responses were observed on growth rate (−12.8 g/day, *P<*0.05) and body composition (+0.9 mm backfat thickness, *P*=0.57; −2.64% lean meat content, *P*<0.001) with a marked response on feed conversion ratio (−0.32 kg feed/kg gain, *P*<0.001). Reduced ultimate pH and increased lightness of the meat (*P*<0.001) were observed in LRFI pigs with minor impact on the sensory quality of the meat. These changes in meat quality were associated with changes of the muscular energy metabolism. Reduced maintenance energy requirements (−10% after five generations of selection) and activity (−21% of time standing after six generations of selection) of LRFI pigs greatly contributed to the gain in energy efficiency. However, the impact of selection for RFI on the protein metabolism of the pig remains unclear. Digestibility of energy and nutrients was not affected by selection, neither for pigs fed conventional diets nor for pigs fed high-fibre diets. A significant improvement of digestive efficiency could likely be achieved by selecting pigs on fibre diets. No convincing genetic or blood biomarker has been identified for explaining the differences in RFI, suggesting that pigs have various ways to achieve an efficient use of feed. No deleterious impact of the selection on the sow reproduction performance was observed. The resource allocation theory states that low RFI may reduce the ability to cope with stressors, via the reduction of a buffer compartment dedicated to responses to stress. None of the experiments focussed on the response of pigs to stress or challenges could confirm this theory. Understanding the relationships between RFI and responses to stress and energy demanding processes, as such immunity and lactation, remains a major challenge for a better understanding of the underlying biological mechanisms of the trait and to reconcile the experimental results with the resource allocation theory.

## Implications

Selection for low residual feed intake (RFI) in growing pigs as a measure of the net feed efficiency is feasible with limited impacts on other production traits and no marked reduction of the pig ability to face challenges, including lactation. Residual feed intake can therefore be used to improve feed efficiency in growing pigs. Indicators to identify efficient sows with high lifetime feed efficiency and longevity are also pointed out. Finally, because no genomic or biomarker was identified, methodologies using direct phenotyping and genomic selection are likely keys of future efficient breeding programmes for feed efficiency.

## Introduction

Selection to improve feed use in livestock remains a challenge in most species. Despite significant improvements in animal genetics and management (e.g. housing, feeding techniques, health), feed cost still represents about two-thirds of the production cost in Western countries (69% in pigs in 2013 in France, IFIP-GTE, 2014). In addition to the economic pressure of feed cost, reducing the environmental impact and diminishing the competition with land use for the production of human food and biofuel are major challenges. Moreover, pigs still contribute 10% to 15% of the nitrogen (N) and phosphorus (P) excretion by livestock in Europe in intensive animal production areas. Swine production generates large needs of manure management and negatively affects the societal image of pig production. Feed efficiency during growth is generally expressed as its inverse trait, the feed conversion ratio (FCR) corresponding to the ratio of inputs (feed intake (FI)) to outputs (BW gain). The FCR can generally be improved indirectly by selection for increased growth rate and decreased adipose tissue. Since the 1990s, single-place electronic feeders have been utilized to record individual feed intake of group-housed pigs in conditions close to commercial breeding, resulting in more accurate and intense selection for feed efficiency. However, selecting for FCR includes associated improvement of efficiency for production traits, such as growth rate and body composition, and of efficiency for other functions that would not directly impact production. As early as 1963, Koch *et al.* (Supplementary Material S1) proposed the concept of RFI, also called net feed efficiency, to specifically capture the efficiency of feed use independent from production needs. The RFI can be computed at the phenotypic or at the genetic level (Supplementary Material S1, Kennedy *et al.*, 1993) as the difference between observed FI and FI predicted for production and maintenance needs. The choice of traits to predict FI for production requirements differs between species and studies, but there is a general agreement that RFI is moderately heritable (Saintilan *et al*., 2013). To evaluate the potential of RFI for the improvement of feed efficiency in pigs, studies based on commercial populations (e.g. Saintilan *et al*., 2013) or experimentally selected lines have been developed in the past 20 years. The establishment of experimental lines is a common strategy to evaluate the direct and correlated responses to a criterion for selection and to study the impact of the selection on animal physiology. The main drawback is the risk of interpreting responses resulting from genetic drift as responses to selection (Supplementary Material S1, Hill, 1972). Two independent sets of divergent lines have considered RFI as a criterion for selection in Large White/Yorkshire growing pigs: one conducted at INRA (Gilbert *et al*., 2007) and one conducted at Iowa State University (ISU; Cai *et al*., 2008). This review proposes an overview of the results from the divergent selection experiment conducted at INRA over 10 generations of selection and surveys the remaining challenges and perspectives for the improvement of feed efficiency in growing pigs.

## Selection experiment for residual feed intake

The selection experiment was conducted in two INRA experimental herds (GenESI, France). The G0 generation was selected from 30 Large White litters (115 male candidates to selection) from 30 sires representing the diversity of the commercial French Large White population and 30 dams (F0). Each line was later managed at each generation with six boars, and 35 to 40 dams distributed in the two herds (Gilbert *et al.,* 2007). In each generation, 96 male candidates were tested per line, and the six with the lowest (LRFI line) or highest (HRFI line) RFI were retained to produce the next generation. An average selection pressure of 7% was applied across generations on males, whereas no selection pressure was applied to the dams. In each generation, one parity was produced to select breeding boars and choose gilts for the next generation. At least one additional parity was produced to evaluate the correlated responses to selection on production traits on both females and castrated males. Data comprised records from 1451 male pig candidates to selection in generations G0 to G8 and records from 1629 females and castrated males to compute the responses to selection from G1 to G9. The average inbreeding level in generation G9 was 0.19 in the LRFI line and 0.18 in the HRFI line.

Animals born in a given farrowing batch were gathered at weaning (28 days of age) in the same post-weaning unit in one farm. They were tested from 10 weeks of age until slaughter (105 kg BW until G5 and 115 kg BW afterwards) in four pens per batch equipped with single-place electronic feeders. In all batches, 12 animals were allotted per pen. Animals were offered *ad libitum* a pelleted diet based on cereals and soya bean meal containing 10 MJ net energy (NE)/kg and 160 g CP/kg, with a minimum of 0.80 g digestible Lys/MJ NE.

The criterion for selection (index) was obtained from phenotypic correlations between daily feed intake (DFI), average daily gain (ADG) and backfat thickness (BFT) at 95 kg BW estimated in an earlier study (Supplementary Material S1, Labroue *et al*., 1999) as DFI (g/day)−(1.06×ADG (g/day))−(37×BFT (mm)). This criterion for selection was used to select future breeding pigs in each generation. Candidates were tested over a fixed BW range (35 to 95 kg BW). The average metabolic BW (AMBW; Supplementary Material S1, Noblet *et al.*, 1999) during the test was thus 12.13 kg^0.60^ for all individuals, and individual variations of maintenance requirements due to differences in AMBW was not accounted for in the index computation. After testing, a second RFI was also computed using realized phenotypic correlations to evaluate the difference between the fixed selection index and the actual performances. This new RFI trait had a genetic correlation of 0.92 with the index. It had a phenotypic standard deviation (*σ*_*p*_) of 132 g/day, its phenotypic correlation with the index was 0.97 and it accounted for 38% of the variability in DFI. On the females and castrated males sibs of the candidates to selection tested to evaluate the responses to selection from 10 weeks of slaughter BW, a test RFI for this extended period was estimated as the residual of a multiple linear regression on DFI accounting for the effects of sex, pen size, contemporary group, BW at beginning of the test, together with AMBW to account for maintenance requirements and ADG during the test and carcass BFT (carcBFT) and lean meat content (LMCcalc; computed from cut weights) at slaughter to account for production requirements. After 10 generations of selection, the multiple linear regression had an *R* ^2^ of 0.75, and the resulting RFI was DFI (g/day)−(1.47×ADG (g/day))+(31.2×LMCcalc (%))–(0.05×carcBFT (mm))−(49.1×AMBW (kg) – fixed effects.

## Genetics of residual feed intake and impacts on main dimensions of pig production

### Responses to selection on production and carcass traits

**Figure 1:**
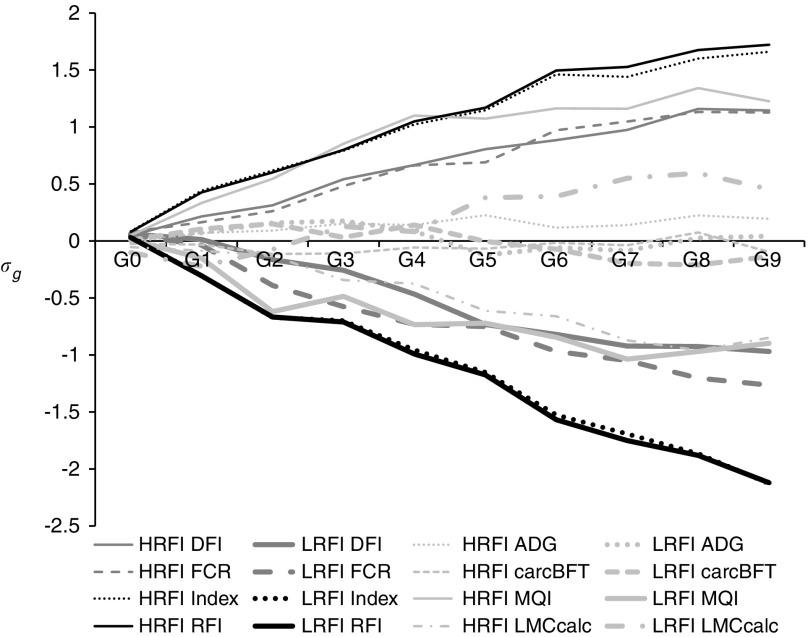
Genetic trends in the divergent selection experiment for residual feed intake (RFI) on component traits and meat quality expressed in genetic standard deviations of the traits (*σ*_*g*_), obtained from a linear mixed model including an animal random effect structured by the pedigree relationship matrix. HRFI=high RFI line; LRFI=low RFI line; Index=selection index; DFI=daily feed intake; ADG=average daily gain; FCR=feed conversion ratio; carcBFT=backfat thickness measured on the carcass; MQI=meat quality index; LMCcalc=lean meat content of the carcass computed from a linear combination of cut weights.

The selection index in the divergent lines had a heritability of 0.13±0.05 (Table 1), just the same as the heritability estimated for RFI computed from the realized phenotypic correlations in the population (i.e. 0.13±0.05). These figures are lower than generally reported for growing pigs (0.20 to 0.40, Saintilan *et al*., 2013). The genetic correlation between RFI and FCR was 0.39±0.12. The responses to selection were significant since G1 on RFI and DFI (Figure 1), reaching −165 g/day (LRFI line – HRFI line) for RFI (3.84 genetic standard deviation (*σ*_*g*_)), and −270 g/day for DFI in generation G9 (Table 1). The line difference for FCR was −0.32 kg feed/kg BW gain. Correlated responses to selection on ADG were slightly significant (−8 g/day) in generation G9, but no clear increase over successive generations was observed. Responses on carcBFT were not significant, but LMCcalc was 1.30 *σ*_*g*_ higher in the LRFI compared with the HRFI line in G9, indicating that predicting RFI from BFT does not fully constrain changes in body composition to achieve better feed efficiency. This was associated with significant increases in dressing percentage, loin, ham and shoulder weights and reductions in backfat and belly weights. In the ISU lines, a significant decrease of ADG was reported in the low RFI line compared with the high RFI line, and similar to the INRA lines, the LRFI pigs had more muscle and less fat (Young and Dekkers, 2012). In addition to differences in DFI, a reduced water intake as g/kg BW^0.60^ per day (−33%, *P*=0.062) was reported in LRFI pigs (Renaudeau *et al*., 2013).

**Table 1:** Genetic parameters (*h* ^2^=heritability; *ρ*_*g*_=genetic correlation with RFI; *σ*_*g*_=genetic SD; *σ*_*p*_=phenotypic SD), responses to selection^1^ in generation G9 at the genetic level in the low RFI (LRFI) and high RFI (HRFI) lines and significance level of the difference (*P*)

Traits	*h* ^2^	*ρ*_*g*_	*σ*_*g*_	*σ*_*p*_	HRFI	LRFI	*P* ^2^
Index (point)	0.13		6.63	18.60	11.00 (0.27)	−14.10 (0.27)	***
RFI (g/day)	0.13		42.93	119.56	73.88 (1.71)	−91.03 (1.71)	***
FCR	0.42	0.39	0.13	0.20	0.15 (0.01)	−0.17 (0.01)	***
DFI (g/day)	0.41	0.25	127.63	199.85	146.22 (6.18)	−123.67 (6.18)	***
ADG (g/day)	0.50	−0.07	54.12	76.45	10.53 (2.71)	2.31 (2.71)	*
DP (%)	0.36	0.05	1.06	1.76	−0.50 (0.05)	0.49 (0.05)	***
Loin weight (kg)	0.54	0.15	0.42	0.57	−0.38 (0.02)	0.33 (0.02)	***
Ham weight (kg)	0.51	0.09	0.32	0.45	−0.24 (0.02)	0.07 (0.02)	***
Shoulder weight (kg)	0.38	0.06	0.28	0.45	−0.15 (0.01)	0.16 (0.01)	***
Backfat weight (kg)	0.43	−0.08	0.30	0.46	0.13 (0.02)	−0.14 (0.02)	***
Belly weight (kg)	0.28	0.11	0.21	0.39	0.23 (0.01)	−0.17 (0.01)	***
LMCcalc (%)	0.59	0.14	2.02	2.64	−1.72 (0.10)	0.92 (0.10)	***
carcBFT (mm)	0.40	0.02	2.40	3.80	−0.23 (0.12)	−0.32 (0.12)	
pH 24 h AD	0.41	0.28	17.06	26.57	14.69 (0.86)	−9.01 (0.86)	***
pH 24 h SM	0.38	0.22	12.03	19.48	12.90 (0.60)	−8.08 (0.60)	***
pH 24 h GS	0.39	0.23	10.84	17.31	12.36 (0.53)	−9.08 (0.53)	***
pH 24 h LM	0.32	0.19	10.41	18.50	9.99 (0.48)	−5.13 (0.48)	***
L* GM	0.20	−0.14	1.59	3.55	−0.24 (0.07)	0.36 (0.07)	***
L* GS	0.33	−0.17	2.13	3.71	−2.27 (0.09)	1.83 (0.09)	***
a*_GM	0.29	−0.09	1.26	2.34	0.05 (0.06)	−0.43 (0.06)	***
a*_GS	0.26	0.12	0.88	1.73	0.09 (0.04)	−0.02 (0.04)	t
b*_GM	0.24	−0.12	0.80	1.65	−0.02 (0.04)	−0.09 (0.04)	
b*_GS	0.32	−0.08	0.84	1.48	−0.55 (0.04)	0.41 (0.04)	***
WHC (s)	0.04	−0.29	10.11	48.22	3.80 (0.32)	−3.11 (0.32)	***
MQI	0.33	0.26	1.61	2.80	1.97 (0.08)	−1.44 (0.08)	***

Index=selection index; FCR=feed conversion ratio; RFI=residual feed intake; DFI=daily feed intake; ADG=average daily gain; LMCcalc=lean meat content of the carcass computed from a linear combination of cut weights; carcBFT=backfat thickness measured on carcass; DP=dressing percentage of cold carcass; pH 24 h: determined 24 h after slaughter; AD=adductor; SM=semimembranosus; GS=*gluteus superficialis*; LM=*longissimus* muscle; GM=*gluteus medius*; L*=lightness, a*=redness, b*=yellowness, measured 24 h after slaughter; WHC=water holding capacity; MQI=meat quality index.

1 Least square means (SE) of a linear model including the fixed effects of line, generation and the interaction line×generation applied to the estimated breeding values of the pigs tested in the two lines from G0 to G9 (*n*=1451 candidates to selection and 1629 slaughtered sibs).

^2^ *P* value for the difference between least square means of the LRFI and LRFI lines in generation G9, ***=*P*<0.001, *=0.01<*P*<0.05, *t*=0.05<*P*<0.10.

### Meat quality traits

The substantial progress made since the 1960s to increase growth rate and carcass LMC and to improve feed efficiency in swine has resulted in unfavourable effects on meat quality (Supplementary Material S1, Lebret, 2004). From the third generation of divergent selection for RFI, technological quality traits and sensorial meat quality indicators were impaired in the LRFI line in both loin (*longissimus*) and ham (*semimembranosus*, *gluteus*, *adductor*) muscles (Tables 1 and 2) (Gilbert *et al*., 2007; Lefaucheur *et al*., 2011; Faure *et al*., 2013). In generation G9, these differences reached −3.41 points (−2.12 *σ*_*g*_) for a meat quality index based on a combination of ultimate pH, L* and water holding capacity. These responses were related to changes in tissue deposition efficiency and in muscle metabolic properties (see *Biological components of residual feed intake* section). Indeed, a higher percentage of fast-twitch glycolytic myofibres and a hypertrophy of all fast-twitch myofibres were found in the *longissimus* muscle (LM) of LRFI pigs, resulting in greater muscle glycogen stores, especially in the glycolytic muscles (Lefaucheur *et al*., 2011). Besides, the intra-muscular fat content was lower in the glycolytic and oxidative muscles of the LRFI line (Lefaucheur *et al*., 2011; Faure *et al*., 2013), without any impact on oxidation traits of lipids and proteins in meat after ageing (Supplementary Material S1, Gilbert *et al*., 2012a). These unfavourable genetic relationships between low RFI and meat quality traits were confirmed in French commercial populations (Saintilan *et al*., 2013), but were not always found in other studies: in the ISU RFI lines, Smith *et al*. (2011) reported no line difference in ultimate pH, drip loss or colour coordinates of loin, but Arkfled *et al*. (2015) reported lower drip loss, colour scores, lean tissue a* and lipid content and greater moisture content in LRFI pigs in later generations of the same selection experiment.

**Table 2:** Meat quality traits of the *longissimus* muscle of pigs from low (*n*=60) and high (*n*=57) residual feed intake lines of generation G6

	Line		
	HRFI	LRFI	*P* ^1^
pH 30 min p.m.	6.42	6.38	Ns
pH 24 h p.m.	5.68	5.59	***
Drip loss (1 to 4 days p.m.) (%)	2.69	3.78	***
Colour			
Lightness (L*)	51.5	52.7	*
Redness (a*)	8.1	8.7	*
Yellowness (b*)	5.0	5.6	*
Intra-muscular fat content (%)	1.39	1.17	**

HRFI=high residual feed intake; LRFI=low residual feed intake; p.m.=*post mortem*. Adapted from Faure *et al*. (2013).

1 Ns=non-significant at *P*>0.05, *P<0.05, **P<0.01, ***P<0.001.

**Figure 2:**
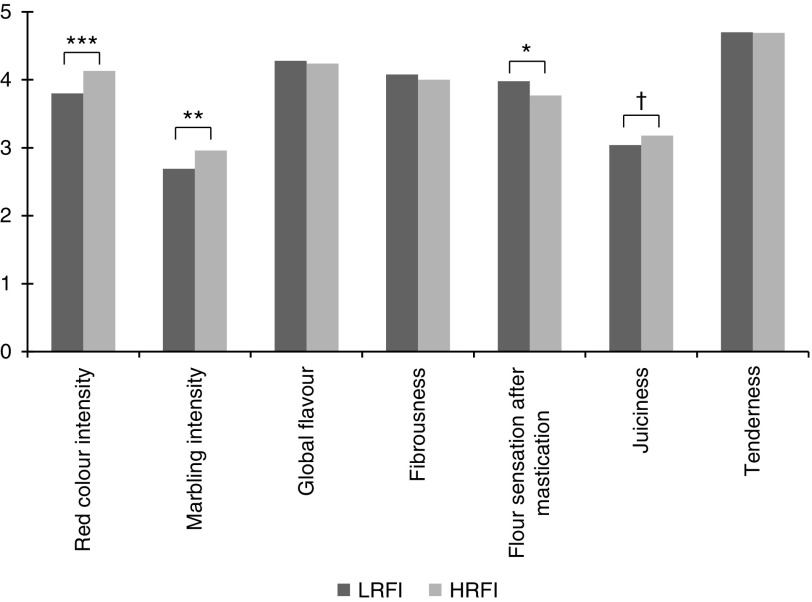
Sensorial meat quality in the loin evaluated on a 0 to 10 scale (none to very high) in pigs from a low residual feed intake (RFI) line (LRFI, *n*=60) and a high RFI line (HRFI, *n*=57) after seven generations of selection. Red colour intensity and marbling intensity were appreciated on raw meat, the other traits were appreciated on cooked meat (dry heat for 10 min at 250°C and then humid heat at 100°C up to a core temperature of 80°C). †0.05<*P*<0.1, *P<0.05, **P<0.01, ***P<0.001. Adapted from Faure *et al*. (2013).

Sensorial analyses in the INRA lines showed that the appearance of raw meat was significantly modified, but eating quality of loin was lowly affected by selection (Figure 2) as reported for the ISU lines (Arkfled *et al*., 2015), despite muscle metabolic responses to selection. Besides, a multidimensional analysis allowed the identification within the LRFI line of a sub-group of efficient pigs that exhibited both lean carcasses and satisfactory levels of technological and sensory meat quality (Supplementary Material S1, Faure *et al.*, 2012).

### Growth and feed intake curves and nutrient requirements

**Figure 3:**
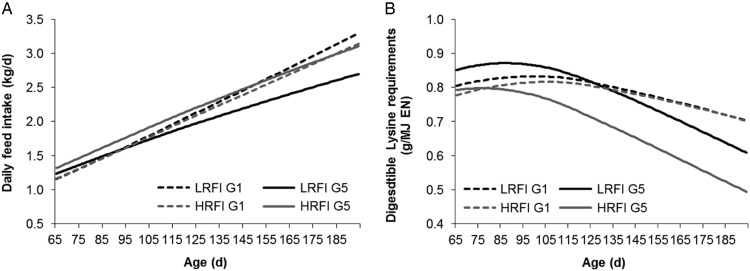
Curves for daily feed intake and digestible lysine requirements during the growing–finishing period of low residual feed intake (LRFI) and high RFI (HRFI) lines in generations G1 and G5 of selection as predicted using the INRAPorc^®^ model (*n*=1370). First, recorded BW were used to fit a Gompertz model to the repeated BW records. Next, the daily feed intake (DFI) records were modelled for each individual with a non-linear exponential model DFI=*a*×BW^*b*^ (Gilbert *et al*., 2009). Finally the daily digestible lysine requirements were calculated individually with InraPorc^®^ on the basis of modelled protein deposition and observed growth and DFI curves as described in Saintilan *et al*. (2015).

Correlated responses to selection for RFI on growth and DFI curves were investigated. Gilbert *et al*. (2009) analyzed individual curves for growth and DFI on the first six generations of selection of the RFI lines to obtain individual lysine requirement curves from the InraPorc^®^ model (Figure 3). Growth rate was slightly lower in LRFI animals throughout the growing period (−7.2% on average, data not shown). The DFI was also lower in LRFI animals (−6% to −13% from early growth to late growth), resulting in higher digestible lysine requirements (in g lysine/MJ NE) during the whole growing period, ranging from +7% at 10 weeks of age up to +20% at slaughter weight. Estimation of genetic parameters with RFI, DFI and shape descriptors of curves describing DFI (e.g. estimated DFI at 50 kg BW, DFI_50_) and growth showed that DFI_50_ could be an interesting early predictor for RFI, having a heritability of 0.41 and a genetic correlation of 0.61 with RFI. Line differences in amino acid requirements were later validated experimentally by feeding all pigs a control diet covering requirements of all pigs or by feeding a diet covering the requirements of the lowest 25% of the HRFI pigs. Feeding the low-lysine diet reduced ADG in the LRFI line to a larger extent than in the HRFI line (−25% *v.* −5%, respectively) (Supplementary Material S1, Brossard *et al.*, 2012). In the ISU RFI lines Cai *et al*. (2011, 2012) also reported that the LRFI line had lower DFI, especially in the later part of the growth period. These results indicate that the dynamics of DFI and possibly growth can respond to selection on RFI over a rather long period (30 to 95 kg). The relationships between RFI and model parameters characterizing growth, DFI and lysine requirements curves were also analyzed in commercial pigs (Saintilan *et al*., 2015), confirming different dynamics depending on feed efficiency. This indicates that the amino acid content of the diet (and probably other nutrients) must be considered when selecting for efficient pigs to avoid poor performance due to mismatch between nutrient supplies and requirements. This will be more important for entire males, which are leaner and more efficient than barrows.

### N and P excretion

Selection for improvement of feed efficiency is expected to reduce the environmental impact through a decrease of N and P excretion, at least by decreasing the total amount of excreta. In the sixth generation of selection for RFI, N and P excretion, evaluated by a modelling approach, was shown to be slightly reduced in LRFI pigs (4.0% and 2.4%, respectively; Supplementary Material S1, Faure *et al.*, 2012). However, some of these differences were lower or not observed in experimental trials during short periods of time where animals were individually housed (Barea *et al*., 2010) or exposed to stressors (Renaudeau *et al*., 2013; Labussière *et al*., 2015). Recent studies on the genetic relationships between feed efficiency and excretion in commercial populations allowed to quantify moderate to high genetic correlations between N excretion and feed efficiency using predictive equations (Table 3; Saintilan *et al*., 2013) or direct measurement (Shirali *et al*., 2012, 2014). Also, Saintilan *et al*. (2013) reported moderate to high correlations between P excretion and feed efficiency in these populations. However, these studies were performed with only one diet fed to the animals, and provide no evidence of differences in efficiency of N or P deposition in relation with RFI. The correlations confirm the potential of the selection for reduced RFI or FCR to decrease excretion when the diet is not adjusted to the pig requirements. Taking into account the differences in nutrient requirements between animals to supply adequate nutrient levels should enhance the effects of RFI improvement. However, possible interactions with feed composition and stress must also be considered when reasoning the impact of RFI on excretion levels.

**Table 3:** Genetic correlations (SE) between feed efficiency traits (feed conversion ratio (FCR); residual feed intake (RFI)) and excretion traits recorded in performance test station in three commercial populations

	RFI			FCR		
Traits	Landrace	Large White	Pietrain	Landrace	Large White	Pietrain
FCR	0.53 (0.07)	0.52 (0.05)	0.85 (0.04)	–	–	–
DFI	0.61 (0.06)	0.55 (0.05)	0.48 (0.09)	0.51 (0.07)	0.37 (0.06)	0.20 (0.11)
ADG	0.07 (0.11)	0.16 (0.08)	−0.05 (0.12)	−0.51 (0.07)	−0.39 (0.06)	−0.42 (0.09)
Ne	0.48 (0.07)	0.46 (0.06)	0.84 (0.04)	0.99 (0.00)	0.99 (0.00)	0.99 (0.00)
Pe	0.52 (0.07)	0.51 (0.05)	0.85 (0.04)	0.99 (0.00)	0.99 (0.00)	0.99 (0.00)
Nr	0.41 (0.07)	0.38 (0.06)	0.83 (0.04)	0.97 (0.01)	0.97 (0.01)	0.98 (0.01)
Pr	0.52 (0.07)	0.52 (0.05)	0.86 (0.04)	0.99 (0.01)	0.99 (0.01)	0.99 (0.01)

Adapted from Saintilan *et al*. (2013). DFI=average daily feed intake; ADG=average daily gain; Ne=N quantity excreted during the test period; Pe=P quantity excreted during the test period; Nr=proportion of retained nitrogen relative to feed intake; Pr=proportion of retained phosphorus relative to feed intake.

### Genomic dissection of residual feed intake

**Figure 4:**
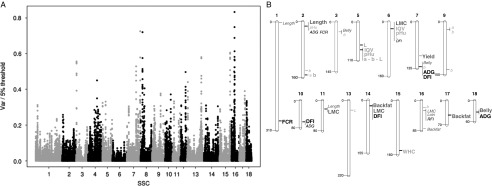
(a) Manhattan plot for detection of associations between SNP and residual feed intake (RFI) (*n*=149 low RFI pigs, *n*=121 high RFI pigs), using a genomic relationship matrix to account for the pedigree structure. The y-axis corresponds to the ratio between the variance explained by successive 0.3 Mb windows as estimated from a single-step genomic selection approach and the empirical 5% significance threshold at the genome level computed by simulation under the null hypothesis. (b) Chromosomal location of SNPs with suggestive (small italic) or significant (large bold) *P* for each group of traits (growth rate, feed intake and feed efficiency in black, carcass traits in dark grey and meat quality traits in light grey). Adapted from Riquet *et al*. (2014). FCR=feed conversion ratio; RFI=residual feed intake; DFI=daily feed intake; ADG=average daily gain; LMC=lean meat content of the carcass computed from a linear combination of cut weights; backfat=backfat thickness measured on carcass or backfat weight; Length=carcass length; Yield=carcass yield; pHu=pH determined 24h after slaughter on *adductor, semimembranosus, gluteus superficialis* or *longissimus* muscles; L=lightness, a=redness, b=yellowness, measured 24 h after slaughter on *gluteus superficialis* or *gluteus medius* muscles; WHC=water holding capacity; MQI=meat quality index.

Even though feed intake can be measured accurately, it is an expensive trait to record. The advances in genomics now allow the identification of genomic regions affecting the variability of quantitative traits (QTL) to consider selection schemes incorporating molecular information as predictors of feed efficiency and related traits. A genome wide association study to detect QTL for RFI and production traits was run using a single-step approach in the selected lines (Riquet *et al*., 2014). All sires from generations G0 to G6 and dams from generations G0, G3 and G6 were genotyped with 60 K single nucleotid polymorphisms (SNPs) (149 LRFI and 121 HRFI). Only few significant regions were identified (Figure 4) for all traits given the limited power of the study and only suggestive regions were identified for RFI: the most significant one was located on *sus scrofa* chromosome (SSC) 16 (Figure 4). Three, two and one regions were detected for DFI, ADG and FCR, respectively. Some of these regions were already detected in other experiments: the QTL detected on SSC7 affecting DFI and ADG could be similar to the QTL influencing the same traits in the ISU lines at position 125 Mb (Onteru *et al*., 2013). The two QTL influencing ADG (SSC7, 130.5 Mb; SSC18, 32.5 Mb) have also been reported by Fontanesi *et al*. (2014). On the opposite, the regions detected on SSC10 and SSC14 for DFI and SSC1 for FCR have not been reported before, neither the suggestive region for RFI on SSC16. A comparative analysis of these candidate regions with bovine results highlighted two suggestive regions on SSC7 and SSC8 orthologous to QTL regions affecting RFI in cattle (*bos taurus* chromosome (BTA)BTA21, 70 Mb: Santana *et al*., 2014 and BTA6, 45 Mb: Rolf *et al*., 2012). These first results are promising, but require confirmation. The absence of a significant region for RFI suggests that biological strategies for improving this trait are diverse. The identification of the sub-traits contributing to RFI could also contribute to the dissection of the trait.

## Biological components of residual feed intake

Lower RFI could result from the improvement of various functions using energy and nutrients, such as improved digestion, more efficient intermediary metabolism, and reduced maintenance and activity requirements. Understanding these processes could lead to the identification of biomarkers to be used as early predictors of feed efficiency in pigs.

### Digestion and fibrous diets

The digestibility was measured at multiple sites of the gastro intestinal tract (GIT) to compare the digestive efficiency of the lines at each step of the digestion process. However, there was no line difference in these digestibility measurements for nutrients and energy during the post-weaning and growing stages in pigs fed conventional European diets (Barea *et al*., 2010; Montagne *et al*., 2014; Labussière *et al*., 2015). Such minor role of digestion in explaining RFI differences has been reported in laying hens and broilers (Supplementary Material S1, Luiting *et al*., 1994) and in mice (Supplementary Material S1, Bunger *et al*., 1998), whereas digestibility has been shown to contribute very significantly to RFI in beef cattle (Herd and Arthur, 2009).

Compared with feeding a control diet, feeding the LRFI and HRFI pigs with a low-energy high-fibre diet supplied by a mixture of wheat bran, dehydrated sugar beet pulp and soya bean hulls during 3 weeks decreased digestibility similarly in both lines, despite lower weights of the digestive tract in LRFI pigs (Montagne *et al*., 2014). The faecal NDF digestibility and concentrations of volatile fatty acids in the caecum were lower in LRFI pigs compared with HRFI pigs when fed the high-fibre diet (Montagne *et al*., 2014). The increase in eating time of pigs fed with a low-energy high-fibre diet was 22% for the HRFI line, whereas it was up to 30% for the LRFI line (Supplementary Material S1, Hauptli *et al.*, 2013). The dietary fibre significantly increased the ratio of acetate to propionate concentrations in the distal part of the GIT for the HRFI line only, which might affect the metabolism of peripheral tissues. The visceral mass is a major source of utilization of nutrients and energy (Supplementary Material S1, Yen *et al.,* 1989), and fermentation in the hindgut diverts nutrients from the animal metabolism. Both were reduced in LRFI pigs, which may contribute to their better efficiency. The reduction of ADG observed when feeding a high-fibre diet during 3 weeks was smaller in the LRFI pigs than in the HRFI pigs (Montagne *et al*., 2014). However, other studies found a similar reduction in ADG in both lines when a high-energy high-fibre diet was given to pigs during 10 weeks (Gondret *et al*., 2014). The LRFI pigs from the ISU lines fed a conventional US diet had a greater digestive efficiency in early generations (Harris *et al*., 2012), which was only found in animals fed a low-energy high-fibre diet in the later generations (Supplementary Material S1, Mauch *et al.*, 2015). Altogether, the digestive efficiency does not seem to explain the variation in RFI between lines. The two lines also show some differences in gut microbiota composition, in particular in the phylotypes of the *Prevotella* and *Lactobacillus* genus present in each line (Zemb *et al*., submitted) that could contribute to their different use of the feed.

### Energy and protein metabolism in healthy animals

Both nutrient use and metabolism in tissues may participate to the biological basis of feed efficiency. Different observations suggest that LRFI pigs have a reduced nutrient catabolism for energy production in skeletal muscles. Indeed, lower glycolytic and lower oxidative enzyme activities have been reported in their muscles (Le Naou *et al*., 2012; Faure *et al*., 2013). These changes were associated with a reduced activity of the adenosine monophosphate-activated protein kinase (AMPK), a sensor of the cellular energy deficit that generally acts as a master switch to stimulate oxidation. Importantly, a reduced oxidation of nutrients to provide ATP later released as heat was found in LRFI pigs (Barea *et al*., 2010; Renaudeau *et al*., 2013; Labussière *et al*., 2015). In the LM of LRFI pigs, lower expression levels of genes associated with mitochondrial metabolism (Vincent *et al*., 2015) have been reported, including several antioxidant proteins. This could indicate a lower oxidative stress: a positive correlation between RFI and reactive oxygen species was also reported in LM mitochondria in ISU lines (Grubbs *et al*., 2013). However, when evaluated in bundles of 20 to 30 fresh permeabilized myofibres, no difference in the potential of mitochondrial respiration or in the expression of genes coding for uncoupling proteins were found between LRFI and HRFI lines in earlier generations of selection (Lefaucheur *et al*., 2011). Because efficiency is directly linked to energy use, muscles of LRFI pigs may have fewer but more efficient mitochondria. Decreased rates of the Cori cycle (involving glucose and lactate turnovers between muscle and liver) may also participate to limit energy losses in the LRFI pigs (Le Naou *et al*., 2012). Paradoxically, in adipose tissues increased mitochondrial oxidative enzyme activities had been reported in LRFI pigs (Gondret *et al*., 2014), a metabolic change that can account for their leaner phenotype. Other molecular changes in adipose tissues concerned many genes involved in the regulation of apoptosis and cell death and immunity pathways (Louveau *et al*., 2016).

It is also reasonable to suspect changes in protein metabolism to account for differences in efficiency between RFI lines. However, due to differences in methodologies, age/weights of studied animals and experimental designs, it is difficult to render definitive conclusions on this aspect. First, there was no difference in nitrogen utilization (as % of absorbed N) between LRFI and HRFI lines, resulting in similar rates of protein deposition in animals from the 6^th^ generation (Barea *et al*., 2010). Nitrogen utilization and protein deposition were also similar between lines in the 7^th^ generation during post-weaning (Labussière *et al*., 2015), but were lower in LRFI animals during the growing period (Renaudeau *et al*., 2013). Second, similar rate of protein synthesis and expressions of protein synthesis markers have been reported in LM in the two lines both at INRA (Le Naou *et al*., 2012) and at ISU (Cruzen *et al*., 2013). On the other hand, Vincent *et al*. (2015) have shown an over-expression of various genes encoding initiation and elongation translation factor subunits in LM of LRFI pigs at market weight. Regarding protein catabolism, the overall proteasome and calpain activities in muscle did not differ between RFI lines when pigs were considered early in the post-weaning period as well as at market weight (Le Naou *et al*., 2012). However, Cruzen *et al*. (2013) have reported that muscles of ISU LRFI pigs at 68 kg BW exhibited lower activities of the 20S proteasome, a key catalytic subunit responsible for proteolysis of ubiquitin-tagged proteins, and of calpains involved in Ca^2+^-dependent proteolysis. Further investigations are thus needed to clarify the possible involvement of protein metabolism in the muscle and the viscera in divergence for RFI.

### Basal metabolism

An important fraction of the difference in energy requirements between LRFI and HRFI lines is related to differences in basal metabolic rate. In growing pigs, basal metabolic rate is generally estimated through the determination of fasting heat production (FHP). In 60 kg BW pigs kept in thermoneutral conditions (24°C) and fed close to *ad libitum*, a modelling approach used to partition total heat production (HP) between its different components indicated that FHP was lower in LRFI pigs than in HRFI pigs (−10% on average, Barea *et al*., 2010), which is consistent with results reported by Boddicker *et al*. (2011) in the ISU lines. Similarly, basal metabolic rate estimated by HP measurements in feed-deprived animals has also been shown to be numerically lower in LRFI laying hens (−15%, Supplementary material S1, Gabarrou *et al*., 1997) and cockerels (Swennen *et al*., 2007). Little information is available in the literature on the physiological mechanisms underlying the lower FHP in LRFI pigs. As in others species (Supplementary Material S1, Bottje *et al*., 2006; Herd and Arthur, 2009; Murphy *et al*., 2013), differences in mitochondria number or activity in tissues could contribute to explain changes in basal metabolic rate between the two lines (see Energy and protein metabolism in healthy animals section). In addition, the variation in energy expenditure and especially the FHP component due to line differences in the size of visceral organs can contribute to the differences in basal metabolic rate between animals with different RFI (see Digestion and fibrous diets section). However the direct effect of a decreased FI only explained 25% of the maintenance difference between the lines (Labussière *et al*., 2015).

### Activity and feeding behaviour

Activity is one of the main non-productive functions contributing to energy use in the pig. The LRFI pigs spent less time standing (Table 4), leading to reduced physical activity (−2.5% of a 24 h scan for time standing, i.e. −35 min, representing a 21% difference in generation G6; Meunier-Salaün *et al*., 2014). This difference represented 14% of the line difference in metabolizable energy (ME) intake (Meunier-Salaün *et al*., 2014), which agrees with calorimetric observations (17%; Barea *et al*., 2010). A similar line effect in physical activity was described in the ISU lines (Sadler *et al*., 2011). Investigations conducted in other species also reported differences in physical activity related to RFI (Supplementary Material S1, Luiting *et al.*, 1994; Supplementary Material S1, Bunger *et al*., 1998; Herd and Arthur, 2009). These differences were not due to a higher prevalence of lameness in LRFI pigs (Meunier-Salaün *et al*., 2014): in fact, higher scores were observed in HRFI pigs, potentially related to their higher physical activity. Social and pen investigations were slightly reduced in LRFI pigs, but feeding activity significantly contributed to differences in physical activity (Table 4): LRFI pigs showed shorter daily eating time, lower number of visits and increased feeding rate (Gilbert *et al*., 2009; Meunier-Salaün *et al*., 2014). In ISU lines, Young *et al*. (2011) reported a trend for LRFI to visit the feeders fewer times and to spend less time eating per visit. These line differences could be modified if the pen composition was modified, for example, by mixing lines or sexes. Investigations of the impact of the selection on the social interactions between individuals have to be explored to complete the evaluation of the activity component.

**Table 4:** Behavioural activity in pigs from the low residual feed intake (LRFI) and high residual feed intake (HRFI) lines, on pigs from generation G6

	Line			
Traits^1^	LRFI	HRFI	RMSE	*P* ^2^
Behavioural activity (% on 24 h)^3^				
Standing	9.6	12.1	0.2	***
Feeding	4.3	5.4	0.1	***
Social investigation	3.2	3.5	0.1	0.08
Pen investigation	3.5	3.9	0.1	0.07
Feeding patterns^4^				
Feed intake (g/day)	1959	2123	272	***
Number of visits/day	13	19	5	***
Feed intake/visit (g)	190	159	52	***
Visit duration (s)	299	270	79	Ns
Feeding rate (g/min)	38	35	1	***

Adapted from Meunier-Salaün *et al*. (2014).

1 Traits in reference to the animal trait ontology for livestock ATOL: http://www.atol-ontology.com/index.php/en/.

2 Ns=non-significant at *P*>0.10, ***P<0.001.

3 Video recordings during 24 h at 17 weeks of age (mean BW 77±8 kg); % on total scan (*n*=96 LRFI, *n*=96 HRFI). Least square means, RMSE and *P* from linear mixed models including the batch, line and sex as fixed effects and interactions between these factors. Analysis was done on the ratio value after arcsin racin transformation.

4 Feeding patterns were determined during a 3 to 4 weeks-period surrounding 12 (P1), 17 (P2) and 22 (P3) weeks of age (*n*=140 LRFI; *n*=125 HRFI). Least square means, RMSE and *P* from linear mixed models including the batch, line, sex and growth stage (P1=54±7 kg; P2=77±8 kg; P3=96±9 kg) as fixed effects, interactions between these factors and the animal as repeated random effect. Only line effects for period P2 are presented.

### Biological markers

Identifying early RFI biomarkers to easily measure large numbers of animals is highly desirable. Among the potential sources of biomarkers, blood is the most frequently targeted biological fluid: it stands as a surrogate tissue that can be repeatedly sampled by minimally invasive procedures. First, circulating concentrations of IGF-I, a growth factor synthesized by the liver and most tissues and known to play a major role in growth and metabolism (Supplementary Material S1, Le Roith *et al*., 2001) have been analyzed. Juvenile IGF-I, that is circulating IGF-I measured soon after weaning, has been shown to have significant genetic correlations with production traits in commercial populations (Supplementary Material S1, Bunter *et al.*, 2005), and to respond to selection for RFI in the ISU lines (Bunter *et al*., 2010). Investigations in the INRA RFI lines confirmed lower circulating juvenile IGF-I concentrations in LRFI pigs compared with HRFI pigs (Bunter K. and Louveau I., unpublished results). This difference was not found at market weight (Lefaucheur *et al*., 2011; Le Naou *et al*., 2012). Second, a lower plasma leptin level in the fed state has been observed in LRFI pigs at 100 kg BW (Lefaucheur *et al*., 2011) and a positive genetic correlation between RFI and serum leptin concentrations has been reported in Duroc pigs (Hoque *et al*., 2009). However, the precise relationships between these blood measurements and RFI deserve further studies.

Advances in high-throughput technologies offer new opportunities to identify novel physiological markers of feed efficiency. Plasma metabolomic profiles did not clearly discriminate 132-day-old pigs from the RFI lines (Jégou *et al*., 2015). By contrast, transcriptomic profiles of whole blood examined between 35 and 132 days of age showed line differences both at INRA (Jégou *et al*., 2016) and at ISU (Supplementary Material S1, Liu *et al*., 2015). The main differences were found in the expression of genes associated with the immune system, despite limited line differences in response to inflammatory and immune challenges (see Stress and other functions: robustness and residual feed intake section). Proteomic analyses may also be a relevant approach to find serum biomarkers of RFI in young pigs (Grubbs *et al*., 2014). Altogether, these putative candidates need to be further validated as relevant biomarkers of feed efficiency.

## Stress and other functions: robustness and residual feed intake

Selection on RFI impacted metabolism and some non-productive functions. Resulting differences in nutrient partitioning might lead to changes in robustness (Knap, 2009; Hermesch *et al*., 2015). Particularly, selection for low RFI may alter the ability to re-allocate nutrients for stress and defence responses when facing environmental challenges. However, 1.8 times less pigs were culled between 10 weeks of age and slaughter in the LRFI line compared with the HRFI line in the seven first generations of selection, suggesting that LRFI pigs might be more robust than HRFI pigs (Supplementary Material S1, Pastorelli *et al*., 2015). In addition, in response to a novel object placed in the pens no line difference could be observed between the INRA lines (Meunier-Salaün *et al*., 2014) and reduced behavioural reactivity was reported in LRFI pigs in the ISU lines (Colpoys *et al.*, 2014). None of these studies suggested a detrimental effect of selection for RFI on pig welfare. Below are reported the detailed responses of the RFI lines to three types of situations classically occurring in pig breeding and involving biological functions that are not directly related to growth: facing an inflammatory challenge, heat stress and lactation.

### Response to an inflammatory challenge

The inflammatory response is caused by sanitary events the pig has to face during his lifespan and is a typical situation inducing trade-offs between productive and non-productive functions. To investigate the line differences in metabolic and immune responses to an inflammatory challenge, weaned piglets were injected with Complete Freund’s Adjuvant (CFA) to induce a non-infectious pneumonia (Supplementary Material S1, Melchior *et al.*, 2004). Both lines showed a similar transient depression in FI during the first 24 h and hyperthermia during the first 2 days. They also displayed similar increases in blood levels of haptoglobin, a hepatic inflammatory protein, and interferon (IFN)-*γ*, an inflammatory cytokine (Merlot *et al*., 2011). Blood transcriptome analysis indicated that few genes related to inflammation and immunity as well as to other functions, including antioxidant defences, proteasome and lysosome functions were differently expressed between lines during the acute phase of the inflammatory challenge. Together with the transcriptome data from whole blood and adipose tissue of older healthy animals from the INRA lines presented above, these results suggest that the differences in the gene profiling could account for baseline differences between the LRFI and HRFI lines rather than for differences in the line capacity to respond to inflammation (Rogel-Gaillard C. and Merlot E., unpublished results). In the tissues where the immune response develops, for example in lungs and their draining lymph nodes, the expression of inflammatory cytokines was lower in LRFI pigs 1 week after the CFA challenge.

The first 2 days after CFA administration, contrary to HRFI piglets, LRFI piglets exhibited a marked reorientation of nutrients from anabolic to catabolic pathways (Labussière *et al*., 2015). On the 8^th^ day, during the recovery phase, differences in the potential for protein accretion were observed between lines (Labussière *et al*., 2015; Merlot *et al*., 2016). The tendency for a higher muscle protein synthesis rate and lower indicators of protein catabolism (muscle calpain activity and plasma hydroxyproline levels), and the higher blood clearance of dietary amino acid (AA) immediately after the meal in LRFI compared with HRFI pigs supports the hypothesis that selection for low RFI favoured the preservation of muscular protein accretion during inflammation (Merlot *et al*., 2016). Divergent selection might have generated preferences for different energetic pathways in LRFI and HRFI pigs to respond to inflammation. Indeed, individual AA plasma concentrations on the week after the CFA challenge suggested that the alanine cycle, producing pyruvate in the liver, might be more reduced in LRFI than in HRFI pigs. To conclude, in young pigs the lines differed moderately in their immune and metabolic responses to an inflammatory challenge, and no disadvantage was observed for the LRFI line. Further studies of chronic immunity and sanitary challenges should provide a complementary understanding of these line responses.

### Response to heat stress

**Figure 5:**
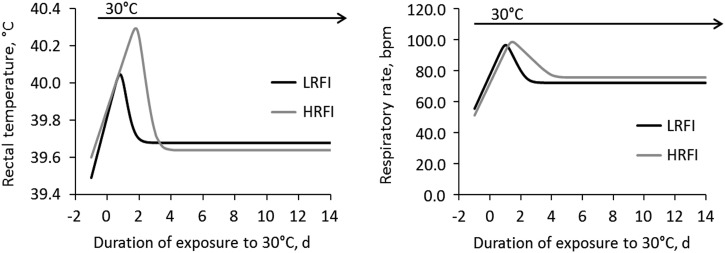
Effects of line and high ambient temperature on rectal temperature and respiratory rate in growing pigs from the low residual feed intake (LRFI) and high residual feed intake (HRFI) lines. The thermoregulatory responses were modelled using the following equation: *Y*=*y*_0_+*v*_1_ *h*−*r*_1_(*v*_1_−*v*_2_) ln{1+exp[(*d*−th_1_)/*r*_1_]}−*r*_2_(*v*_2_−*v*_3_) ln{1+exp[(*d*−th_2_)/*r*_2_]} where *Y* is the response variable; *d* the day of exposure, *y*_0_ the value of *Y* on day 0 *L*; th_1_ and th_2_ the threshold days of the first and second phase of response, respectively; *v*_1_, *v*_2_ and *v*_3_ are the linear variations of *Y* before and after th_1_ and th_2_, respectively. Only the th_1_ parameter for rectal temperature was significantly affected by the line (0.85 *v.* 1.88 day, for the LRFI and the HRFI line, respectively). Adapted from Campos *et al*. (2014).

In hot conditions, the capacity to maintain homoeostasis is driven by the animal’s ability to lose heat and/or to reduce its metabolic HP. Selection for low RFI reduced the total amount of heat produced by unit of ME intake (see Basal metabolism section), which could favour LRFI pigs to cope with high temperatures. In a first approach, thermoregulatory responses and energy utilization in HRFI and LRFI lines were compared during a standardized thermal challenge (Renaudeau *et al*., 2008). Although not significant, LRFI pigs had a numerically lower reduction of FI in hot conditions than HRFI pigs (Campos *et al*., 2014). In addition, the time required to initiate acclimation responses was shorter in LRFI compared with HRFI pigs (Figure 5). However, this favourable low RFI line effect was not accompanied by changes in blood hormones concentration and/or in the ability to lose heat (Renaudeau *et al*., 2013; Campos *et al*., 2014). In the former study, changes in energy metabolism during the thermal acclimation period at 32°C were similar in both lines. In connection with their higher water intake (see Responses to selection on production and carcass traits section), HRFI pigs tended to have a greater capacity to lose heat by evaporation in hot and thermoneutral conditions that could compensate their greater HP. Similar results were reported in HRFI laying hens (Supplementary Material S1, Bordas and Minvielle, 1997). In a second approach, the RFI lines have been evaluated in a tropical environment. From 11 to 23 weeks of age, the ADG was slightly lower in the LRFI than in the HRFI. This effect was mainly explained by a reduced growth of the LRFI line during the post-weaning period without a compensation during the growing–finishing period (Supplementary Material S1, Gilbert *et al*., 2012b).

### Lactation

**Figure 6:**
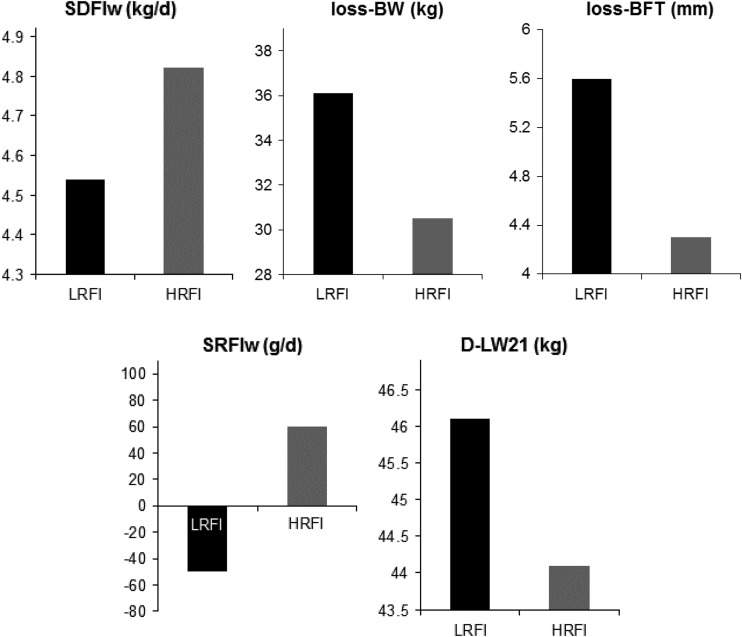
Correlated responses to selection for residual feed intake (RFI) after seven generations of divergent selection for sow reproductive traits. LRFI=low RFI line; HRFI=high RFI line; SDFI=sow daily feed intake; loss-BW=body weight loss during lactation; loss-BFT=backfat thickness loss during lactation; D-LW21=litter weight gain from farrowing to 21 days of age; SRFI=sow residual feed intake during lactation. All line differences were significant at *P*<0.001. Adapted from Gilbert *et al*. (2012).

Decreased voluntary FI during growth combined with increased growth rate, leanness and prolificacy limits the availability of sow resources to face lactation, which is the only period of negative energy balance in healthy pigs, with potential deleterious impacts on reproductive performance and longevity (Prunier *et al*., 2010). Despite low genetic correlations between RFI and sow reproductive traits (Gilbert *et al*., 2012), the correlated responses to selection after seven generations of selection (Figure 6) showed significantly reduced daily FI during lactation (−280 g/day) in the LRFI line compared with the HRFI line, increased loss of BW and BFT (+5.6 kg BW and +1.3 mm BFT), higher litter weight at 21 days of age (+2 kg), and +0.6 weaned piglet, but no change in piglet BW at weaning (28 days of age) despite numerically heavier piglets in most studies in the LRFI line. Given the low number of unsuccessful inseminations, no significant line difference was reported for rebreeding. Young *et al.* (Supplementary Material S1, 2010) obtained similar results at the phenotypic level in sows of the ISU lines after seven generations of selection. Applying the RFI concept to lactating females (Supplementary Material S1, Veerkamp *et al.*, 1995) on the INRA sows showed a reduction of 110 g/day of the lactation RFI after seven generations of selection in LRFI sows (Gilbert *et al*., 2012), indicating that LRFI sows are more resource-efficient for a given production level. When assessed in breeding sows from the eighth generation in tropical conditions (Renaudeau *et al*., 2014), the line difference in voluntary FI during lactation was further increased (−590 g/day in the LRFI line compared with the HRFI line), as were BW and BFT losses, probably essentially due to the combination of heat and humidity. Altogether, the two experiments show no deleterious impact of selection for low RFI during growth on sow reproduction traits, even when FI is restricted by heat stress. This can be interpreted as an improved capacity of LRFI sows to produce milk and ensure litter survival compared with HRFI sows, suggesting a higher robustness of the LRFI sows. The dynamics of body resources and FI during lactation and gestation over multiple parities in LRFI pigs could provide indicators of life production efficiency of the sows.

## Conclusion

**Figure 7:**
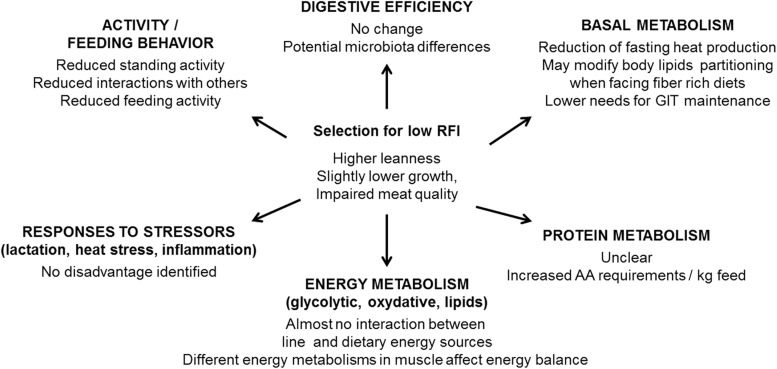
Impacts of the reduction of residual feed intake (RFI) on major physiological functions in growing pigs. GIT=gastro intestinal tract, AA=amino acid.

The impacts of 15 years of divergent selection for RFI on growing pigs raised under conventional controlled conditions, but also on alternative situations are summarized in Figure 7. These results confirmed the potential of RFI for selection for feed efficiency. In addition, our results support that selection for low RFI does not compromise the ability of the animals to face challenges. However, strategies to incorporate this trait into selection schemes remain to be studied. It has been suggested that the linear combination of the components of RFI, already present in most selection objectives, should be sufficient, but proper calculation of the economic weight of RFI remains to be elucidated. For the selection of feed efficiency, phenotyping FI, or predicting accurately RFI via proxies or markers, is a second major challenge that affects RFI as well as FCR. Despite numerous studies, neither genetic markers nor blood biomarkers have been reported yet, but the recently developed techniques of genomic prediction might provide a solution of choice for such a polygenic trait.

In addition, the absence of improvement of digestive efficiency in response to selection for RFI indicates that improving this function could only be obtained when challenging the pigs with dietary fibre during the selection process (Noblet *et al.*, 2013). The gut microbiota might also play a role in this improvement. Finally, the absence of unfavourable relationships between RFI and responses to stress, which is also observed in the ISU lines, remains a challenge to explain and is not consistent with the resource allocation theory. In particular, the unexpected good lactation performance of the LRFI sows should be further studied to identify profiles of highly efficient lactating sows that are able to rebuild their body reserve after lactation and mobilize them when needed, ensuring longevity.

## Supplementary material

For supplementary material accompanying this paper visit http://dx.doi.org/10.1017/S175173111600286X. click here to view supplementary material
