# Supplementary material for: Review: divergent selection for residual feed intake in the growing pig
Source: Animal. 2017 Jan 25;11(9):1427–39. doi: 10.1017/S175173111600286X (PMC5561440; doi:10.1017/S175173111600286X)
Supplement: Supplementary file 1 [file S175173111600286Xsup001.docx]

Divergent selection for residual feed intake in the growing pig: a review

H. Gilbert, Y. Billon, L. Brossard, J. Faure, P. Gatellier, F. Gondret, E. Labussière, B. Lebret, L. Lefaucheur, N. Le Floch, I. Louveau, E. Merlot, M.-C. Meunier-Salaün, L. Montagne, P. Mormede, D. Renaudeau, J. Riquet, C. Rogel-Gaillard, J. van Milgen, A. Vincent and J. Noblet

SUPPLEMENTARY REFERENCES

Bordas A and Minvielle F 2000. Réponses à la chaleur de poules opndeuses issues de lignées sélectionnées pour une faible (R^–^) ou forte (R^+^) consummation alimentaire résiduelle. Genetic Selection Evolution 29, 279-290.

Bottje W, Pumford NR, Ojano-Dirain C, Iqbal M and Lassiter K 2006. Feed efficiency and mitochondrial function. Poultry Science 85, 8-14.

Brossard L, Gilbert H, Billon Y and van Milgen J 2012. Effet d’une sélection divergente pour la consommation journalière résiduelle chez le porc en croissance sur la réponse à une carence en acides aminés. Journées de la Recherche Porcine 44, 165-170.

Bunger L, MacLeod MG, Wallace CA and Hill WG 1998. Direct and correlated effects of selection for food intake corrected for BW in the adult mouse. In Proceedings of the 6th World Congress on Genetics Applied to Livestock Production, 11-16 January 1998, Armidale, Australia, pp. 97-100.

Bunter KL, Hermesch S, Luxford BG, Graser HU and Crump RE 2005. Insulin-like growth factor-I measured in juvenile pigs is genetically correlated with economically important performance traits. Australian Journal of Experimenatl Agriculture 45, 783-792.

Faure J, Lebret B, Brossard L, Billon Y, Lefaucheur L and Gilbert H 2012. Vers une sélection sur la consommation alimentaire résiduelle chez le porc en croissance pour concilier efﬁcacité alimentaire, qualité des viandes et impact environnemental. Journées de la Recherche Porcine 44, 25-30.

Gabarrou JF, Geraert PA, Picard M and Bordas A 1997. Diet-induced thermogenesis in cockerels is modulated by genetic selection for high or low residual feed intake. Journal of Nutrition 127, 2371-2376.

Gilbert H, Bidanel JP, Billon Y, Méteau K, Guillouet P, Noblet J, Sellier P, Gatellier P, Sayd T, Faure J and Lebret B 2012a. Responses to divergent selection for residual feed intake in growing pigs, consequences on pork. In Book of Abstracts of the 63th Annual Meeting of the European Association for Animal Production, 27-31 August 2012, Bratislava, Slovakia, pp. 276.

Gilbert H, Billon Y, Fleury J, Noblet J, Gourdine JL and Renaudeau D 2012b. Are responses to selection in lines divergently selected for residual feed intake in growing pigs affected by GxE interactions when bred in a tropical environment? In Proceedings of the AnGR-NordicNET Workshop, Genotype-by-Environment Interactions and Adaptation of Farm Animals on Phenotypic and Molecular Levels, 7-8 November 2012, Tuusula, Finnland, pp. 26-27.

Hauptli L, Priet A, Gilbert H and Montagne L 2013. Response to a high level fibers diet in pigs divergently selected for residual feed intake. In Book of Abstracts of the 64th Annual Meeting of the European Association for Animal Production, 26-30 August 2013, Nantes, France, pp. 589.

Hill WG, 1972. Estimation of genetic change. I. General theory and design of control populations. Animal Breeding Abstracts 40, 1–15.

Kennedy BW, van der Werf JHJ and Meuwissen THE 1993. Genetic and statistical properties of residual feed intake. Journal of Animal Science 71, 3239–3250.

Koch RM, Swiger LA, Chambers D and Gregory KE 1963. Efﬁciency of feed use in beef cattle. Journal of Animal Science 22, 486–494.

Labroue F, Guéblez R, Meunier-Salaün MC and Sellier P 1999. Feed intake behaviour of group-housed Piétrain and Large White growing pigs. Annales de Zootechnie 48, 247–261.

Lebret B 2004. Rationalization of pig production: consequences on meat quality. Productions Animales 17, 79-91.

Le Roith D, Bondy C, Yakar S, Liu JL and Butler A 2001. The somatomedin hypothesis. Endocrine Reviews 22, 53-74.

Liu H, Nguyen Y, Nettleton D, Dekkers JCM and Tuggle CK 2015. Identification of early blood differentially expressed genes between two pig lines divergently selected for feed efficiency: potential biomarkers for feed efficiency. In Proceedings of the 48th Annual Meeting of the ASAS Midwestern Section and the ADSA® Midwestern Branch, 16–18 March 2015, Des Moines, USA, pp. 106.

Luiting P, Urff EM and Verstegen MWA 1994. Between-animal variation in biological efficiency as related to residual feed consumption. Netherlands Journal of Agricultural Science 42, 59–67.

Mauch ED, Gabler NK, Weber TE, Patience JF, Kerr BJ and Dekkers JCM 2015. The effect of divergent selection for residual feed intake on digestibility of control and low energy, high fiber diets. In Proceedings of the 48th Annual Meeting of the ASAS Midwestern Section and the ADSA® Midwestern Branch, 16–18 March 2015, Des Moines, USA, pp. 46.

Melchior D, Seve H and Le Floc'h N 2004. Chronic lung inflammation affects plasma amino acid concentrations in pigs. Journal of Animal Science 82, 1091-1099.

Noblet J, Karege C, Dubois S and van Milgen J 1999. Metabolic utilisation of energy and maintenance requirements in growing pigs: effect of sex and genotype. Journal of Animal Science 77, 1208–1216.

Pastorelli H, Prunier A, Brossard L, Montagne L, Gilbert H, Larzul C and Merlot E 2015. Evaluation of relations between health, death and growth in growing pigs. Journées de La Recherche Porcine 47, 57-62.

Veerkamp RF, Emmans GC, Cromie AR and Simm G 1995. Variance components for residual feed intake in dairy cows. Livestock Production Science 41, 111–120.

Yen JT, Nienaber JA, Hill DA and Pond WG 1989. Oxygen consumption by portal vein-drained organs and by whole animal in conscious growing swine. Proceedings of the Society for Experimental Biology and Medicine 190, 393-398.

Young JM, Bergsma R, Knol EF, Patience JF and Dekkers JCM 2010. Effect of selection for residual feed intake on sow reproduction performance and lactation efficiency. In Proceedings of the 9th World Congress on Genetics Applied to Livestock Production, 1-6 August 2010, Leipzig, Germany, abstract #223.
